# Interaction Effect of Subjective Health and Attitude about Aging on Gerontechnology Acceptance

**DOI:** 10.1093/geroni/igab046.2486

**Published:** 2021-12-17

**Authors:** Hayoung Park, Susanna Joo, Kwang Joon Kim, Chang Oh Kim, Yun Mook Lim, Hey Jung Jun

**Affiliations:** 1 Yonsei University, Seoul, Seoul-t'ukpyolsi, Republic of Korea; 2 Yonsei University, Seodaemun-gu, Seoul-t'ukpyolsi, Republic of Korea; 3 Severance Hospital, Yonsei University College of Medicine, Seoul, Seoul-t'ukpyolsi, Republic of Korea

## Abstract

This study examined the interaction effect of subjective health and attitude about aging on gerontechnology acceptance among Korean older adults. The sample was 310 Korean older adults aged 65 and above without cognitive impairment who completed an online survey. The dependent variable was the attitude about gerontechnology, especially an exoskeleton robot for exercise. The independent variable was subjective health measured by the questions about self-reported health conditions. The moderating variable was the attitude about aging, which was measured by asking how much they feel less useful as they age. Covariates were age, gender, education level, employment status, income, and marital status. The results from the regression analyses using PROCESS macro and bootstrapping showed that the interaction effect of subjective health and attitude about aging is significant for gerontechnology acceptance; when older adults consider themselves less useful as they age, they tend to have positive attitudes about gerontechnology despite their subjective health. However, of those who consider that they are not less useful as they age, they tend to have positive attitudes about gerontechnology only when they consider themselves unhealthy. Thus, they tend to have negative attitudes about gerontechnology when they consider themselves healthy and useful. The findings imply that gerontechnology-based exercise programs or interventions could be welcomed by those who consider themselves unhealthy and think they are useless as they age. Also, the findings suggest that when applying an intervention program using gerontechnology, health status and self-assessment of aging should be considered in advance.

